# Parents' Hesitancy to Vaccinate Their 5–11-Year-Old Children Against COVID-19 in Saudi Arabia: Predictors From the Health Belief Model

**DOI:** 10.3389/fpubh.2022.842862

**Published:** 2022-03-30

**Authors:** Ohoud S. Almalki, Osamah M. Alfayez, Majed S. Al Yami, Yousif A. Asiri, Omar A. Almohammed

**Affiliations:** ^1^Department of Clinical Pharmacy, College of Pharmacy, Taif University, Taif, Saudi Arabia; ^2^Department of Pharmacy Practice, College of Pharmacy, Qassim University, Qassim, Saudi Arabia; ^3^Department of Pharmacy Practice, College of Pharmacy, King Saud bin Abdulaziz University for Health Sciences, Riyadh, Saudi Arabia; ^4^Department of Clinical Pharmacy, College of Pharmacy, King Saud University, Riyadh, Saudi Arabia; ^5^Pharmacoeconomics Research Unit, College of Pharmacy, King Saud University, Riyadh, Saudi Arabia

**Keywords:** vaccine, COVID-19, hesitancy, parents, Health Belief Model, Saudi Arabia

## Abstract

Data exploring parents' hesitancy to vaccinate their 5–11-year-old children against COVID-19, and associated factors, is limited. This study aims to investigate parents' beliefs and intentions to vaccinate their 5–11-year-old children using the Health Belief Model in Saudi Arabia. A national, cross-sectional, questionnaire-based study was conducted in November, 2021. The self-administered online questionnaire was distributed to a random sample of parents. Adult parents with at least one 5–11-year-old child were included. The main outcome was parents' intention to vaccinate their 5–11-year-old children. Variability in parents' intention was assessed by demographics, COVID-19-related factors, children's health status, and constructs from the Health Belief Model. Univariate and multivariable logistic regression were used to investigate each factor and adjust for the intervariable effect on parental intention to vaccinate their children. Of the 4,135 participants, 61.9% were hesitant to vaccinate their 5–11-year-old children. Parents aged 31 to 40 years (OR = 1.23; 95% CI, 1.02–1.49) and females (OR = 1.52; 95% CI, 1.25–1.84) had higher odds of being hesitant to vaccinate their children than parents from other groups. Parents who perceived low benefit from the vaccine (OR = 16.3; 95% CI, 12.1–21.9) or who had safety or efficacy concerns (OR = 3.76; 95% CI, 3.10–4.58) were among the most hesitant to vaccinate their children. In conclusion, vaccine hesitancy is prevalent among parents of 5–11-year-old children in Saudi Arabia and those who had beliefs of minimal benefits or lack of safety from the COVID-19 vaccine were more hesitant. Government efforts must be directed toward increasing parents' vaccine awareness and tackling the constructs of the Health Belief Model through a well-designed vaccination campaign.

## Introduction

In light of the reported COVID-19-related hospitalizations and deaths in both children and adolescents, countries that have reached a 70% COVID-19 vaccination rate among their adult populations are now focusing on children as the target population for vaccination (1, 2). Although the impact of the COVID-19 pandemic on children is milder than the impact on adults, many children were infected and developed severe symptoms, which in some cases left them with long term complications (3). Different vaccines with proven effectiveness in preventing COVID-19 associated illness and hospitalization in adults are now available in multiple countries (4). The evidence on the safety and efficacy of some of these vaccines in children 5–11 years old is still evolving. However, these are limited by small sample sizes, and the lack of long-term follow-up to assess safety and efficacy in this vulnerable population (5).

In June 2021, the Saudi Ministry of Health (MoH) extended its COVID-19 vaccination plans to recommend that children >12 years receive the Pfizer-BioNTech and Moderna vaccines (6). This recommendation came after studies published positive safety and efficacy results for these two vaccines among 12–18-year-old children (7, 8). More than 90% of 12–18-year-old children attending school were vaccinated against COVID-19 by September 2021 (9). Later on, the Pfizer-BioNTech COVID-19 vaccine was shown to be safe and efficacious in children 5–11 years of age (10); and on November 3, 2021, the Saudi Food and Drug Authority (SFDA) granted emergency use approval for use in this population (11). However, the Saudi MoH did not offer it to children 5–11 years of age until February 2022, and their vaccination is not mandatory, which is the case for 12–18-year-old children and adults in Saudi Arabia (SA).

In spite of global efforts to promote COVID-19 vaccination, vaccine hesitancy, defined as a “delay in acceptance or refusal of vaccination despite the availability of vaccination services,” may interfere with the success of current pandemic-control efforts (12). The COVID-19 vaccine hesitancy was reported in several countries including the developed countries such as the United States (US) (13, 14). However, studies show that some of the Middle Eastern countries had the highest rates of COVID-19 vaccine hesitancy among adults (15–17). Factors associated with vaccination hesitancy include uncertainty about the benefits of vaccination; fear of side effects; inconvenience or lack of access to the vaccine; the type of vaccine available; and religious beliefs (14, 18).

Parental COVID-19 vaccine hesitancy is occurring worldwide, ranging from 2.8% in Brazil, to 35.2% in Qatar and 42.8% in Bangladesh (18–22). A small (*n* = 333) single center cross-sectional study was conducted in SA to assess the willingness of parents to vaccinate children <18 years of age and found hesitancy in 27% of respondents (23). Another study assessed parental hesitancy about COVID-19 vaccine, and approximately 45% of parents were hesitant about having their children vaccinated against COVID-19 (24). However, all of the above-mentioned studies were limited by small sample size and lack of generalizability to our specific population due to the inclusion of children from different age groups.

It is worth noting that vaccination hesitancy is subject to change over time. A study conducted in the US reported a change in vaccine hesitancy over a six-month period among adult participants. While 46% of participants were hesitant to receive the COVID-19 vaccine in October 2020, this dropped to 35.2% in March 2021 (25). However, this change in behavior does not come with time only. As the Health Belief Model (HBM) suggests that a person may change his/her unhealthy behavior if he/she perceives the risk of having the illness and the severity of its consequences, and the benefit from changing the unhealthy behavior outweigh the barriers to the change. In addition, the model proposes that one's perception of the ability to complete the action of interest and the external factors in one's environment can affect one's desire for a change of unhealthy behaviors (26–28).

In spite of the recent approval of a COVID-19 vaccine for use in 5–11-year-olds and before mandating vaccination for children in this category, we need to assess parents' intention to vaccinate their children to know the barriers or challenges that will be faced when carrying on vaccination campaigns and determine the factors that needs to be emphasized in these campaigns. Thus, this study was conducted to investigate parents' beliefs and intentions about vaccinating their 5–11-year-old children using the HBM in SA.

## Materials and Methods

### Study Design and Subjects

A national, cross-sectional, questionnaire-based study was conducted in SA to assess parents' hesitancy about vaccinating their 5–11-year-old children against COVID-19. The association between parent demographics and COVID-19 related factors as well as constructs from the HBM were also assessed. Parents, or legal guardians, with at least one child between the ages of 5 and 11 years who were attending a pre- or elementary school were eligible to participate in the study. A self-administered online questionnaire was distributed to a random sample of eligible parents from different regions in SA in collaboration with the Ministry of Education (MoE). The Scientific Research Ethical Committee at Taif University reviewed and approved the study design and survey (Approval No. 43-015).

### Data Collection

The MoE selected few elementary schools from different cities in the Central, Northern, Eastern, Western and Southern regions of SA; provided schools' administrators with the study information and asked them to send the invitation for the study along with a link to the online questionnaire to all parents of children in these schools. The schools' administrators sent the link to all parents of children in their schools through the MoE database which has all parent or legal guardian contact information. Parents or legal guardians received text messages with an invitation to the study and a link to the Arabic online questionnaire. The authors of the study had no role in selecting the schools nor had a communication with the schools' administrators or the parents. Parents or legal guardians received text messages with an invitation to the study and a link to the Arabic online questionnaire. Data was collected from November 1 to 30, 2021 at about the time when the Pfizer-BioNTech COVID-19 vaccine was approved for 5–11-year-olds in SA.

### Questionnaire

Data were collected using a questionnaire that consisted of three main sections with close-ended questions. The original and English-translated questionnaires are provided in the Supplementary Materials. The first section [7 items: 1 – 7] collected parent demographics, including age, gender, marital status, and education. The second section [9 items: 8 – 16] investigated history of infection with SARS-CoV-2 among adults and children in the household, the current vaccination status of participants and any of their 12–18-year-old children, and the participants' main source of information about the COVID-19 vaccine. This section also asked participants to indicate the general health status of their children, existence of any chronic health conditions, and receipt of the seasonal influenza vaccine. Participants were then asked about their intention to vaccinate their 5–11-year-old children using a four-level Likert scale that ranged from absolutely yes to absolutely no. The last section [14 items: 17 – 30] focused on concepts from the HBM, including parents' perception of their children's susceptibility to SARS-CoV-2 infection [perceived susceptibility: 3 items] and the severity of their condition if they had the infection [perceived severity: 3 items], parental perception of the benefits of their children receiving the COVID-19 vaccine [perceived benefit: 2 items] and reasons that may prevent them from vaccinating their children [perceived barriers: 4 items], and the motives that would prompt them to vaccinate their children [cues to action: 2 items]. Responses to these items were recorded on a four-point Likert scale, ranging from strongly agree to strongly disagree.

While items in the first two sections were general demographic or health status items or were specifically developed for this study, items in the third section were adopted with minor modifications from a previous study that was translated from English to Arabic and validated for use among the adult population in SA (29). The author was contacted and approved the use of these items. Other items in the questionnaire were reviewed by the authors and three additional researchers to ensure face validity and the questionnaire was piloted among 400 participants to ensure item clarity, validity, and consistency. The questionnaire was then developed and distributed using Google Forms®.

### Statistical Analysis

Descriptive statistics, frequencies with percentage (%), were used to describe variables in the study. Responses to the intention to vaccinate 5–11-year-olds were then pooled into two meaningful categories, definitely yes with probably yes, to represent positive intention, and probably no with absolutely no, to represent negative intention (hesitancy). Responses to items from the HBM were also pooled into two categories: agree (agree with strongly agree) and disagree (disagree with strongly disagree).

Univariate logistic regression was used to investigate the effect of demographics, COVID-19 related factors, 5–11-year-old specific factors, and HBM items, on differences in participants' intention to vaccinate their 5–11-year-old children (the dependent variable in the logistic regression). Significant factors from the univariate analyses were included in a multivariable logistic regression to adjust for intervariable effects on parental intention to vaccinate their children. Results from both models were reported as unadjusted and adjusted, respectively, with odds ratio (OR) and 95% confidence intervals (95% CI). All statistical analyses were performed at a significance level of α <0.05 and conducted using SAS statistical software version 9.4 (SAS institute, Cary, NC, USA).

## Results

### Participant Characteristics

From November 1 to 30, 2,021, 4,135 parents or caregivers from different regions of SA participated in the study. Overall, 81% of the participants were female, 13.4% were non-Saudi residents, and 52.1% were between the age of 31 and 40 years. Most participants reported that they and their 12–18-year-old children received at least two doses of the COVID-19 vaccine. Participant characteristics, SARS-CoV-2 infection, vaccination status for both participants and their 12–18-year-old children, and general health status, existence of any chronic health conditions, and usual receipt of seasonal influenza vaccine for participants' 5–11-year-old children are shown in Tables 1, 2.

**Table 1:** Participant characteristics.

**Characteristics**	**Number (%)**
**Age**	
18–30 years	691/4,135 (16.7)
31–40 years	2,154/4,135 (52.1)
41–50 years	1,092/4,135 (26.4)
51–60 years	156/4,135 (3.8)
> 60 years	42/4,135 (1.0)
**Gender**	
Male	784/4,135 (19.0)
Female	3,351/4,135 (81.0)
**Nationality**	
Saudi	3,583/4,135 (86.6)
Non-Saudi	552/4,135 (13.4)
**Marital status of the caregiver**	
Married	3,859/4,135 (93.3)
Divorced/ Separated	156/4,135 (3.8)
Single	76/4,135 (1.8)
Widow	44/4,135 (1.0)
**Highest level of education**	
< high school	662/4,135 (16.0)
High school	1,253/4,135 (30.3)
Associate degree	276/4,135 (6.7)
Bachelor's degree	1,742/4,135 (42.1)
Postgraduate degree	202/4,135 (4.9)
**Geographic region**	
Central	1,018/4,135 (24.6)
Northern	1,326/4,135 (32.1)
Eastern	914/4,135 (22.1)
Western	560/4,135 (13.5)
Southern	317/4,135 (7.7)
**Annual income**	
< $22,400	2,025/4,135 (49.0)
$22,401–$38,400	1,093/4,135 (26.4)
$38,401–$57,600	604/4,135 (14.6)
$57,601–$80,000	218/4,135 (5.3)
> $80,000	195/4,135 (4.7)
**Main source of information regarding COVID-19**	
Social media	1,566/4,135 (37.9)
Ministry of health resources	1,542/4,135 (37.3)
Television or radio	671/4,135 (16.2)
Health care providers	103/4,135 (2.5)
Friends or family	73/4,135 (1.8)
Others	180/4,135 (4.3)

*COVID-19, coronavirus disease of 2019*.

**Table 2:** Participants' and their children's history of COVID-19 infection, COVID-19 vaccination status for participants and their 12–18-year-old children, and health status of 5–11-year-old children.

**Characteristics**	**Number (%)**
**Infection status of adults in the house**	
Yes	1,570/4,135 (38.0)
No	2,565/4,135 (62.0)
**Vaccination status of the adult participant (parent or caregiver)**	
Received at least two doses	3,713/4,135 (89.8)
Received only one dose	247/4,135 (6.0)
Did not receive any COVID-19 vaccine	154/4,135 (3.7)
Excused population (did not receive any COVID-19 vaccine)	21/4,135 (0.5)
**Infection status of** ** <18-year-old children in the house**	
Yes	889/4,135 (21.5)
No	3,246/4,135 (78.5)
**Vaccination status of 12–18-year-old children**	
Received at least two doses	2,206/2,416 (91.3)
Received only one dose	73/2,416 (3.0)
Did not receive any COVID-19 vaccine	85/2,416 (3.5)
Excused population (did not receive any COVID-19 vaccine)	52/2,416 (2.2)
**General health status of 5–11-year-old children**	
Very good	2,871/4,135 (69.4)
Good	991/4,135 (24.0)
Fair	232/4,135 (5.6)
Poor	26/4,135 (0.6)
Very poor	15/4,135 (0.4)
**Existence of chronic health conditions in 5–11-year-old children**	
Yes	298/4,135 (7.2)
No	3,785/4,135 (92.8)
**History of annual influenza vaccination for 5–11-year-old children**	
Yes	1,050/4,135 (25.4)
No	3,085/4,135 (74.6)

*COVID-19, coronavirus disease of 2019*.

### Vaccine Hesitancy

Overall, 61.9% of participants were hesitant to vaccinate their 5–11-year-old children. After controlling for significant factors, 31–40-year-old participants were slightly more hesitant to vaccinate their children than parents from other age groups [OR = 1.23; 95% CI, 1.02–1.49]. Likewise, females and participants from the southern or western regions of SA were more hesitant to vaccinate their children than males [OR = 1.52; 95% CI, 1.25–1.84] and participants from the central region of SA [OR = 1.34; 95% CI, 1.01–1.80 for the southern]. In addition, married participants were more hesitant to vaccinate their children compared to single participants in the study [OR = 2.42; 95% CI, 1.44–4.04]. Compared to parents with < high-school level of education, parents with education level more than high-school, especially those with associate degree, were more hesitant to vaccinate their children. The association between participant demographics and intent to vaccinate 5–11-year-old children is presented in Table 3.

**Table 3:** Association between participant demographics and their intention to vaccinate their 5–11-year-old children.

**Characteristics**	**Vaccination intention [n (%)]**		**Unadjusted analysis** ^**a**^		**Adjusted analysis^**b**^**
	**Positive**	**Negative**	***p*-value**	**OR (95% CI)**	**OR (95% CI)**
**Numbers**	1,577/4,135 (38.1)	2,558/4,135 (61.9)	———————	———————	———————
**Age**			**<0.0001**		
18–30 years	288/691 (41.7)	403/691 (58.3)		1	1
31–40 years	773/2,154 (36.1)	1,381/2,154 (63.9)		**1.28 (1.07–1.52)**	**1.23 (1.02–1.49)**
41–50 years	409/1,092 (37.5)	683/1,092 (62.5)		1.19 (0.98–1.45)	1.20 (0.97–1.49)
51–60 years	86/156 (55.1)	70/156 (44.9)		**0.58 (0.41–0.83)**	0.70 (0.48–1.04)
> 60 years	21/42 (50.0)	21/42 (50.0)		0.72 (0.38–1.33)	0.82 (0.41–1.65)
**Gender**			**<0.0001**		
Male	385/784 (49.1)	399/784 (50.9)		1	1
Female	1,192/3,351 (35.6)	2,159/3,351 (64.4)		**1.75 (1.49–2.05)**	**1.52 (1.25–1.84)**
**Nationality**			**0.0098**		
Saudi	1,339/3,583 (37.4)	2,244/3,583 (62.6)		1	1
Non-Saudi	238/552 (43.1)	314/552 (56.9)		**0.79 (0.66–0.94)**	0.89 (0.73–1.09)
**Marital status of the caregiver**			**0.0026**		
Single	44/76 (57.9)	32/76 (42.1)		1	1
Married	1,448/3,859 (37.5)	2,411/3,859 (62.5)		**2.29 (1.45–3.63)**	**2.42 (1.44–4.04)**
Divorced/ Separated	67/156 (42.9)	89/156 (57.1)		**1.83 (1.05–3.18)**	1.62 (0.88–2.98)
Widow	18/44 (41.0)	26/44 (59.0)		1.99 (0.93–4.22)	1.78 (0.79–4.06)
**Highest level of education**			**<0.0001**		
< high school	311/662 (47.0)	351/662 (53.0)		1	1
High school	510/1,253 (40.7)	743/1,253 (59.3)		**1.29 (1.07–1.56)**	**1.28 (1.04–1.56)**
Associate degree	94/276 (34.1)	182/276 (65.9)		**1.72 (1.28–2.30)**	**1.76 (1.29–2.39)**
Bachelor degree	587/1,742 (33.7)	1,155/1,742 (66.3)		**1.74 (1.45–2.09)**	**1.55 (1.27–1.89)**
Postgraduate degree	75/202 (37.1)	127/202 (62.9)		**1.50 (1.09–2.07)**	**1.47 (1.04–2.08)**
**Regions**			**0.0003**		
Central	397/1,018 (39.0)	621/1,018 (61.0)		1	1
Eastern	397/914 (43.4)	517/914 (56.6)		**0.83 (0.69–0.99)**	**0.78 (0.64–0.95)**
Western	184/560 (32.9)	376/560 (67.1)		**1.30 (1.05–1.62)**	**1.33 (1.06–1.68)**
Southern	126/317 (39.7)	191/317 (60.3)		0.97 (0.75–1.25)	**1.34 (1.01–1.80)**
Northern	473/1,326 (35.7)	853/1,326 (64.3)		1.15 (0.97–1.37)	1.16 (0.96–1.39)
**Annual income**			0.7829		
< $ 22,400	783/2,025 (38.7)	1,242/2,025 (61.3)		1	———————
$22,401–$38,400	401/1,093 (36.7)	692/1,093 (63.3)		1.09 (0.93–1.27)	———————
$38,401–$57,600	229/604 (37.9)	375/604 (62.1)		1.03 (0.86–1.24)	———————
$57,601–$80,000	88/218 (40.4)	130/218 (59.6)		0.93 (0.70–1.24)	———————
> $80,000	76/195 (39.0)	119/195 (61.0)		0.98 (0.73–1.33)	———————
**Main source of information regarding COVID-19**			0.1145		
Television or radio	258/671 (38.5)	413/671 (61.5)		1	———————
Social media	569/1,566 (36.3)	997/1,566 (63.7)		1.10 (0.90–1.32)	———————
Ministry of health resources	615/1,542 (39.9)	927/1,542 (60.1)		0.94 (0.78–1.13)	———————
Health care providers	47/103 (45.6)	56/103 (54.4)		0.74 (0.49–1.13)	———————
Friends or family	29/73 (39.7)	44/73 (61.3)		0.95 (0.58–1.55)	———————
Others	59/180 (32.8)	121/180 (67.2)		1.28 (0.91–1.81)	———————

*OR, odds ratio; COVID-19, coronavirus disease of 2019. Values in bold represent significant differences from the univariate and multivariable logistic regression.*

*^a^The ORs for the unadjusted analysis are from the univariate logistic regression analyses.*

*^b^The ORs for the adjusted analysis are from the multivariable logistic regression analysis including significant factors from the univariate analyses for factors from this table and Table 4; namely age, gender, educational achievement, nationality, marital status, region, income, infection and vaccination status for adults, and the general health status and annual influenza vaccination for children (5–11 years)*.

Parents who reported receiving one or two doses of the COVID-19 vaccine were less hesitant to vaccinate their 5–11-year-old children against COVID-19 [one dose: OR = 0.55; 95% CI, 0.33–0.92; two doses: OR = 0.36; 95% CI, 0.24–0.56] than parents who were not vaccinated. The history of having COVID-19 infection among adults in the household was another predictor for hesitancy to vaccinate children living with these adults (*p*-value=0.002). Interestingly, the study found an inverse correlation between parents' perceived level of general health status of their children and their intention to vaccinate their children. Parents who perceived their children's general health to be very good were the least hesitant to vaccinate their children whereas parents who perceived their children general health status to be very poor were the most hesitant to vaccinate their children [58.9 vs. 93.3%; OR = 11.6 95% CI, 1.50–90.3]. In addition, those who reported that their 5–11-year-old children received the annual influenza vaccine were less hesitant than parents who did not [OR = 0.46; 95% CI, 0.39–0.53]. The association between participants' COVID−19-related factors and parental intention to vaccinate their 5–11-year-old children against COVID-19 is shown in Table 4.

**Table 4:** Association between participants' COVID-19 related factors and their intention to vaccinate their 5–11-year-old children.

**Characteristics**	**Vaccination intention [n (%)]**		**Unadjusted analysis** ^**a**^		**Adjusted analysis^**b**^**
	**Positive**	**Negative**	***p*-value**	**OR (95% CI)**	**OR (95% CI)**
**Numbers**	1,577/4,135 (38.1)	2,558/4,135 (61.9)		———————	———————
**Infection status of adults in the house**			**0.0020**		
No	1,025/2,565 (36.9)	1,540/2,565 (60.1)		1	1
Yes	552/1,570 (35.2)	1,018/1,570 (64.8)		**1.23 (1.08–1.40)**	**1.19 (1.04–1.37)**
Vaccination status of the adult participant		**<0.0001**			
Did not receive any COVID-19 vaccine	29/154 (18.8)	125/154 (81.2)		1	1
Received only one dose	70/247 (28.3)	177/247 (71.7)		**0.59 (0.36–0.96)**	**0.55 (0.33–0.92)**
Received at least two doses	1470/3713 (39.6)	2243/3713 (60.4)		**0.35 (0.23–0.53)**	**0.36 (0.24–0.56)**
Excused population (did not receive)	8/21 (38.1)	13/21 (61.9)		**0.38 (0.14–0.99)**	0.70 (0.24–2.06)
**Infection status for** ** <18-year-old children in the house**			0.3094		
No	1,251/3,246 (38.5)	1,995/3,246 (61.5)		1	———————
Yes	326/889 (36.7)	563/889 (63.3)		1.08 (0.93–1.26)	———————
**Vaccination status of 12–18-year-old children (*****n*** **=** **2416)**			0.3212		
Did not receive any COVID-19 vaccine	26/85 (30.6)	59/85 (69.4)		1	———————
Received only one dose	29/73 (39.7)	44/73 (60.3)		0.67 (0.35–1.30)	———————
Received at least two doses	897/2,206 (40.7)	1,309/2,206 (59.3)		0.64 (0.40–1.03)	———————
Excused population (did not receive)	22/52 (42.3)	30/52 (57.7)		0.60 (0.29–1.23)	———————
**General health status for 5–11-year-old children**			**<0.0001**		
Very good	1,181/2,871 (41.1)	1,690/2,871 (58.9)		1	1
Good	352/991 (35.5)	639/991 (64.5)		**1.27 (1.09–1.47)**	**1.21 (1.03–1.41)**
Fair	41/232 (17.7)	191/232 (82.3)		**3.26 (2.31–4.60)**	**3.26 (2.28–4.67)**
Poor	2/26 (7.7)	24/26 (92.3)		**8.39 (1.98–35.5)**	**8.62 (2.00–37.1)**
Very poor	1/15 (6.7)	14/15 (93.3)		**9.78 (1.29–74.5)**	**11.6 (1.50–90.3)**
**Existence of chronic health condition in 5–11-year-old children**			0.5677		
No	1,468/3,837 (38.3)	2,369/3,837 (61.7)		1	———————
Yes	109/298 (36.6)	189/298 (63.4)		1.07 (0.84–1.37)	———————
**History of annual influenza vaccination for 5–11-year-old children**			**<0.0001**		
No	1,011/3,085 (32.8)	2,074/3,085 (67.2)		1	1
Yes	566/1,050 (53.9)	484/1,050 (46.1)		**0.41 (0.36–0.48)**	**0.46 (0.39–0.53)**

*OR, odds ratio; COVID-19, coronavirus disease of 2019. Values in bold represent significant differences from the univariate and multivariable logistic regression.*

*^a^The ORs for the unadjusted analysis are from the univariate logistic regression analyses.*

*^b^The ORs for the adjusted analysis are from the multivariable logistic regression analysis including significant factors from the univariate analyses for factors from Table 3 and this table; namely age, gender, educational achievement, nationality, marital status, region, income, infection and vaccination status for adults, and the general health status and annual influenza vaccination for children (5–11 years)*.

All variables from the HBM were significantly associated with differences in participants' intention to vaccinate their 5–11-year-old children. Parents who perceived that their 5–11-year-old children were at high risk for SARS-CoV-2 infection (perceived susceptibility), or severe disease (perceived severity), were less hesitant to vaccinate them than those who did not perceive a high risk for infection or severe disease. In addition, parents who perceived minimal benefits from the COVID-19 vaccine had higher odds of being hesitant to vaccinate their children [OR = 16.3; 95% CI, 12.1–21.9]. Those who had concerns about the safety or efficacy of the COVID-19 vaccines (perceived barriers) also had higher odds of being hesitant to vaccinate their children [OR = 3.76; 95% CI, 3.10–4.55]. Luckily, the cues to action items revealed some promising findings. As among parents who initially reported that they will not vaccinate their children (*n* = 2,558), 48.3% indicated that they will register their children to receive the vaccine if they received adequate amount of information about the vaccine, and 54.4% indicated that they will register their children to receive the vaccine if it was taken by many in the public. The association between parental perception, based on the HBM constructs, and intention to vaccinate their 5–11-year-old children is summarized in Table 5.

**Table 5:** Association between variables from the Health Belief Model and parents' intention to vaccinate their 5–11-year-old children.

**The concepts and variables from the HBM**	**Responses**	**Vaccination intention [n (%)]**		**Unadjusted analysis^**a**^**	**Adjusted analysis^**b**^**
		**Positive**	**Negative**	**OR (95% CI)**	**OR (95% CI)**
**Numbers**		1,577/4,135 (38.1)	2,558/4,135 (61.9)	———————	———————
**Perceived susceptibility**					
The chance of my children getting the SARS-CoV-2 in the next few months is high.	Agree	755/1,543 (48.9)	788/1,543 (51.1)	1	1
	Disagree	822/2,592 (31.7)	1,770/2,592 (68.3)	**2.06 (1.81–2.35)**	**2.09 (1.75–2.50)**
I am worried about the likelihood of my children getting the SARS-CoV-2.	Agree	1,096/2,500 (43.8)	1,404/2,500 (56.2)	1	1
	Disagree	481/1,635 (29.4)	1,154/1,635 (70.6)	**1.87 (1.64–2.14)**	**1.75 (1.45–2.09)**
Getting the SARS-CoV-2 is a possibility for my children.	Agree	1,090/2,541 (42.9)	1,451/2,541 (57.1)	1	1
	Disagree	487/1,594 (30.55)	1,107/1,594 (69.45)	**1.70 (1.50–1.95)**	**1.90 (1.58–2.29)**
**Perceived Severity**					
In general, complications from the SARS-CoV-2 are serious.	Agree	1,320/3,145 (42.0)	1,825/3,145 (58.0)	1	1
	Disagree	257/990 (26.0)	733/990 (74.0)	**2.06 (1.76–2.42)**	**2.10 (1.68–2.64)**
If one of my children gets infected with the SARS-CoV-2, he will be very sick.	Agree	956/2,185 (43.8)	1,229/2,185 (56.2)	1	1
	Disagree	621/1,950 (31.8)	1,329/1,950 (68.2)	**1.67 (1.47–1.89)**	**1.71 (1.43–2.05)**
I am worried that my children will get the SARS-CoV-2.	Agree	1,302/3,136 (41.5)	1,834/3,136 (58.5)	1	1
	Disagree	275/999 (27.5)	724/999 (72.5)	**1.87 (1.60–2.18)**	**1.72 (1.39–2.13)**
**Perceived Benefit**					
Vaccination is a good idea because I will not have to worry about my children catching the SARS-CoV-2.	Agree	1,448/2,413 (60.0)	965/2,413 (40.0)	1	1
	Disagree	129/1,722 (7.5)	1,593/1,722 (92.5)	**18.5 (15.2–22.5)**	**15.2 (11.9–19.9)**
Vaccination decreases the chance of my children getting the SARS-CoV-2 or its complications.	Agree	1,483/2,649 (56.0)	1,166/2,649 (44.0)	1	1
	Disagree	94/1,486 (6.3)	1,392/1,486 (93.7)	**18.8 (15.1–23.5)**	**16.3 (12.1–21.9)**
**Perceived Barriers** ^**c**^					
The possible side effects from receiving the COVID-19 vaccine would interfere with my children's usual activities.	Disagree	852/2,914 (29.2)	2,062/2,914 (70.8)	1	1
	Agree	725/1,221 (59.4)	496/1,221 (40.6)	**3.54 (3.08–4.07)**	**2.95 (2.44–3.57)**
I am concerned about the efficacy of the COVID-19 vaccine.	Disagree	910/2,953 (30.8)	2,043/2,953 (69.2)	1	1
	Agree	667/1,182 (56.4)	515/1,182 (43.6)	**2.91 (2.53–3.34)**	**2.53 (2.09–3.06)**
I am concerned about the safety of COVID-19 vaccine.	Disagree	802/2,917 (27.5)	2,115/2,917 (72.5)	1	1
	Agree	775/1,218 (63.6)	443/1,218 (36.4)	**4.61 (4.00–5.32)**	**3.76 (3.10–4.55)**
I am concerned about the fake/faulty COVID-19 vaccine.	Disagree	776/2,628 (29.5)	1,852/2,628 (70.5)	1	1
	Agree	801/1,507 (53.2)	706/1,507 (46.8)	**2.71 (2.37–3.09)**	**2.15 (1.80–2.58)**
**Cues to Action**					
I will register my children to receive the COVID-19 vaccine, if I was given adequate information.	Agree	1,495/2,731 (54.7)	1,236/2,731 (45.3)	1	1
	Disagree	82/1,404 (5.8)	1,322/1,404 (94.2)	**19.5 (15.4–24.7)**	**14.8 (10.9–20.2)**
I will register my children to receive the COVID-19 vaccine, if the vaccine is taken by many in the public.	Agree	1,400/2,793 (50.1)	1,393/2,793 (49.9)	1	1
	Disagree	177/1,342 (13.2)	1,165/1,342 (86.2)	**6.61 (5.56–7.88)**	**5.31 (4.20–6.71)**

*OR, odds ratio; SARS-CoV-2, Severe acute respiratory syndrome coronavirus type 2; COVID-19, coronavirus disease of 2019. Values in bold represent significant differences from the univariate and multivariable logistic regression.*

*^a^The ORs for the unadjusted analysis are from the univariate logistic regression analyses.*

*^b^The ORs for the adjusted analysis are from the multivariable logistic regression analysis including significant factors from the univariate analyses for factors from Tables 3, 4; namely age, gender, educational achievement, nationality, marital status, region, income, infection and vaccination status for adults, and the general health status and annual influenza vaccination for children (5–11 years).*

*^c^The results for the items under the perceived barriers construct are inversed to unify the interpretation of the OR with the other constructs; as agreement with these statements represents negative perceptions about the vaccine*.

## Discussion

This study was conducted to explore the association between parents' beliefs about the COVID-19 vaccines and their intention to vaccinate their 5–11-year-old children against COVID-19. As of November 2021, more than 60% of parents in SA were hesitant to vaccinate their 5–11-year-old children despite the SFDA approval for use of the Pfizer-BioNTech vaccine for this population. Particular demographics, including gender, COVID-19 infection, and vaccination status, in addition to beliefs about the risk of infection for their children and expected benefits and risks from vaccination, were associated with parental intention to vaccinate their 5–11-year-old children. Moreover, about half of parents who were hesitant to vaccinate their children indicated that they will vaccinate them if provided with adequate information about the vaccine or when the vaccine was taken by many in the public.

In SA, vaccine hesitancy among parents of children 12–18 years of age (23, 24, 30) was much lower than vaccine hesitancy among parents of 5–11-year-old children (27 – 45% vs. 61.9%, respectively). Regional and international studies reported lower rates in some areas, including 35% in Qatar and 33% in Chicago (18, 20), however, these percentages included parents with children >11 years of age, which may explain the differences (18, 20, 23, 24, 30). A preprint from Israel including parents with 5–11-year-old children, similar to our population, reported that 43% of these parents were hesitant to vaccinate their children (31). In July 2021, a small study conducted in Arkansas found that around 30% of parents will not vaccinate their children or only vaccinate them when required; interestingly, the hesitancy rates among parents of 12–18-year-old children were similar to the rate among parents with children younger than 12 years of age (28 and 27%, respectively) (32). Whereas recent studies from other countries found lower rates of vaccine hesitancy, studies from China, Vietnam, and Italy reported that about 26%, 21%, and 18% of parents were hesitant to vaccinate their 5–17-year-old, 3–17-year-old, and 12–18-year-old children, respectively (33–35). These results indicate a declining trend in parents' hesitancy toward the vaccine. Although a trend toward higher hesitancy was noticed when parents of children younger than 12 years were included in the studies, the results among the countries were not consistent which indicate a variation among countries in the actual rate of hesitancy.

Data from the COVID-19 Vaccine Monitor of Kaiser Family Foundation (KFF) were consistent with our findings. They showed that in October 2021, only 27% of parents were willing to vaccinate their 5–11-year-old children, similar to 34 and 26% reported in September and July of 2021, respectively. While parental hesitancy to vaccinate 12–18-year-old children decreased from more than 60% in April 2021 to 41% in September 2021 (36). Parents may be more hesitant to vaccinate younger children due to concerns about long-term safety of these vaccines in this group, especially given that most have not reached adolescence. The concerns among parents on the effect of the vaccines on their children need to be investigated further in post-marketing surveillance studies. The results of these needed studies should then be reported to the public in the vaccination campaigns to address the safety and effectiveness concerns among the public, which were found in the study to be significant predictors for vaccine hesitancy among parents.

Parents who were 31–40 years of age were more hesitant (~64%) to vaccinate their 5–11-year-old children than were parents ≤ 30 years or >50 years of age (~58 and 45%, respectively). These results are consistent with other local and international data associating older parental age with less hesitancy to vaccinate their children (19, 24). While other local studies did not find gender to be a significant predictor of vaccine hesitancy, female participants in this study were considerably more hesitant about having their children vaccinated than their male counterparts (23, 24). This may translate to the fact that negative believes and intentions from parents in the age category of 31–40 years and mothers toward the COVID-19 vaccine might negatively affect parents' decisions to vaccinate their children, which will delay reaching the targeted level of herd immunity. Therefore, to have a successful vaccination campaign for 5–11-year-old children, we need to focus more on educating females and parents in this age category, among other factors. While another local study did not find parents' level of education to be a significant predictor of vaccine hesitancy (23), parents with more than high school education in this study were more hesitant to vaccinate their children than those with < a high school education. This also contradict results from other studies associating low level of parental education with greater hesitancy to vaccinate older children (19, 24). These differences may be explained by the small size and limited representation of participants in these studies, in addition to the wider age group of the vaccine eligible children.

Parent uptake of at least one dose of the COVID-19 vaccine and decision to vaccinate their 5–11-year-old children with the annual influenza vaccine were positive predictors of parents' intention to vaccinate their children with the COVID-19 vaccine. This is similar to what has been reported both locally and internationally (18, 20, 23, 24). Thus, stressing these points when campaigning for the children vaccines may encourage other parents to vaccinate their children; this can be stressed by providing stories of COVID-19 survivals especially those who needed hospitalization or critical care. Although, the existence of chronic health conditions in children was not a significant predictor of vaccine hesitancy, as reported previously (20), parents' intention to vaccinate their 5–11-year-old children was inversely correlated with their perception of these children's general health status, highlighting parent concerns about children with poor health. This underlines the importance of providing parents with adequate information about the safety and efficacy of the COVID-19 vaccines, especially among parents of children with poor health.

While all HBM constructs were significant in predicting parents' intention to vaccinate their 5–11-year-old children, parents' perceived benefits and barriers about the COVID-19 vaccine were among the most important factors. As the model suggests, parents who perceived minimal benefit from the vaccine and higher barriers were more hesitant to vaccinate their children. This finding mirrors that from other studies citing lack of information about the vaccine and concern about vaccine safety or efficacy as the underlying reasons why parents did not vaccinate their children (23, 37). However, findings from previous studies were limited by the small sample size, low representation of the general population, and inclusion of parents with children >11 years of age (23, 37). In addition, our finding indicates that providing parents who were hesitant to vaccinate their children with adequate information about the COVID-19 vaccine would encourage about half of them to vaccinate their children as well as vaccinating many in the public would encourage them to vaccinate their children.

The results of this study may be used to guide the design of an effective vaccination campaign by focusing on the significant predictors identified here. Findings indicated that parents primarily sought information about COVID-19 vaccines from social media and MoH platforms, thus MoH should consider using these platforms of media for its vaccination campaigns. When designing vaccination campaigns, participating parties should focus on delivering information that will minimize perceived barriers while increasing perceived benefits in order to reduce parents' hesitancy about having their children vaccinated. As the HBM suggest, successful vaccination campaigns are ones that are tailored to the public needs determined here by the predictors of parents' hesitancy to vaccinate their children. Moreover, the prevalent level of hesitancy among parents in SA may decline with the conduction of a public health campaign that focus on delivering adequate level of information to parents and the roll-out of vaccination for 5–11-year-old children would encourage hesitant parents to vaccinate their children, as our results indicate.

The study has a few limitations and several strengths. The cross-sectional nature of the study and the reliance on self-reported data does not permit tracking participants' final decisions to vaccinate their children. The online questionnaire method we used does not allow counting the number of invitations sent to parents which hinder our ability to estimate the response rate in the study. Thus, it is not clear if the group that did not participate in the study had different results from the cohort who participated, which may result in selection bias. However, having a large sample of participants from different regions in SA and the lack of direct communication between the authors and the school administrators should have mitigated the effect of any possible selection bias in the study. In addition, the over-representation of females in the study might be due to the higher involvement in their children's education and the existence of their information as the main point of contact in their children's schools. Since females were more hesitant than males in vaccinating their children and those were over represented in the study, this means our results might overestimate the real hesitancy rate. However, it is worth noting that the hesitancy rate among males in our study (~51%) was higher than the highest rate reported in previous studies investigating parents' hesitancy to vaccinate children (23, 24, 30), including children >11 years of age. The timing of the study reflects parents' hesitancy rate in November 2021, which is right after the approval of the vaccine in young children and before it became available for this population to receive it. Thus, it may not reflect the actual hesitancy now after it become available for this population and will not reflect the hesitancy rate if it became mandatory.

Besides that, data were collected near the time when the COVID-19 vaccine was first approved for 5–11-year-old children and before the MoH recommendation that this age group be vaccinated. This allows for true baseline parental hesitancy to be estimated without the need to assume vaccine approval or adjust for the impact of the vaccination campaigns on parents' intention to vaccinate their 5–11-year-old children. The study focused on parents of 5–11-year-old children while other studies estimated vaccine hesitancy among parents of all children <18 years of age. In addition, collaboration with the MoE allowed for a large and nationally representative sample which permits generalization of these findings and provides the MoH with evidence to better plan for its vaccination campaigns.

## Conclusion

Despite the COVID-19 vaccine's approval for use in children aged 5–11 years old at the time of the study, parental hesitancy was prevalent compared to that previously reported for parents of children under 18 years old, which might affect governmental efforts' success in containing the pandemic. Since the social media and MoH platforms were trusted sources among parents to have COVID-19 vaccine related information, the use of these channels of communication to educate the public may facilitate the process. Moreover, efforts should be made to communicate adequate amount of information about the safety and efficacy of the COVID-19 vaccines to parents that are more likely to be hesitant will be crucial to successfully reach the needed level of herd immunity in SA and the return to normal life.

## Data Availability Statement

The raw data supporting the conclusions of this article will be made available by the authors, without undue reservation.

## Ethics Statement

The studies involving human participants were reviewed and approved by Scientific Research Ethical Committee at Taif University. The Ethics Committee waived the requirement of written informed consent for participation.

## Author Contributions

OSA and OAA: conceptualization and project administration. OAA: methodology, software, data curation, formal analysis, and funding acquisition. OSA and MA: validation and writing—original draft preparation. OSA, OMA, and MA: investigation. YA, OMA, and OAA: writing—review and editing. OMA, MA, and YA: visualization. OSA, YA, and OAA: supervision. All authors have read and agreed to the published version of the manuscript.

## Funding

This research was funded by the Researcher Supporting Project [RSP-2021/77], King Saud University, Riyadh, Saudi Arabia.

## Conflict of Interest

The authors declare that the research was conducted in the absence of any commercial or financial relationships that could be construed as a potential conflict of interest.

## Publisher's Note

All claims expressed in this article are solely those of the authors and do not necessarily represent those of their affiliated organizations, or those of the publisher, the editors and the reviewers. Any product that may be evaluated in this article, or claim that may be made by its manufacturer, is not guaranteed or endorsed by the publisher.

## References

[1] Live COVID−19 Vaccination Tracker. Available online at: https://covidvax.live/ (accessed November 23, 2021).

[2] Delahoy MJ Ujamaa D Whitaker M O'Halloran A Anglin O Burns E et al. Hospitalizations associated with COVID−19 among children and adolescents – COVID–NET, 14 states, March 1, 2020–August 14, 2021. MMWR Morb Mortal Wkly Rep. (2021) 70:1255–60. 10.15585/mmwr.mm7036e2 34499627 PMC8437052

[3] Woodruff RC Campbell AP Taylor CA Chai SJ Kawasaki B Meek J et al. Risk factors for severe COVID−19 in children. Pediatrics. (2021) 149: e2021053418. 10.1542/peds.2021-053418 PMC9213563 34935038

[4] Tregoning JS Flight KE Higham SL Wang Z Pierce BF. Progress of the COVID−19 vaccine effort: viruses, vaccines and variants versus efficacy, effectiveness and escape. Nat Rev Immunol. (2021) 21:626–36. 10.1038/s41577-021-00592-1 34373623 PMC8351583

[5] She J Liu l Liu W. Providing children with COVID−19 vaccinations is challenging due to lack of data and wide–ranging parental acceptance. Acta Paediatr. 111:35–44. 10.1111/apa.16137 34614260 PMC8653137

[6] Saudi, MoH,. MOH Begins Vaccinating 12–18 Age Group with Pfizer Vaccine. Available online at: https://www.moh.gov.sa/en/Ministry/MediaCenter/News/Pages/News-2021-06-27-008.aspx (accessed November 23, 2021).

[7] Frenck RW Klein NP Kitchin N Gurtman A Absalon J Lockhart S et al. Safety, immunogenicity, and efficacy of the BNT162b2 Covid−19 vaccine in adolescents. N Engl J Med. (2021) 385:239–50. 10.1056/NEJMoa2107456 34043894 PMC8174030

[8] Ali K Berman G Zhou H Deng W Faughnan V Coronado-Voges M. Evaluation of mRNA−1273 SARS–CoV−2 vaccine in adolescents. N Engl J Med. (2021) 385:2241–51. 10.1056/NEJMoa2109522 34379915 PMC8385554

[9] Godinho, V,. Covid−*19: Over 90% of Students Aged 12 and Over in Saudi Arabia are Vaccinated*. Available online at: https://gulfbusiness.com/covid$-$19--over$-$90--of--students--aged$-$12--and--over--in--saudi--arabia--are--vaccinated/ (accessed November 23, 2021).

[10] Walter EB Talaat KR Sabharwal C Gurtman A Lockhart S Paulsen GC et al. Evaluation of the BNT162b2 Covid−19 vaccine in children 5 to 11 years of age. N Engl J Med. (2021) 386:35–46. 10.1056/NEJMoa2116298 34752019 PMC8609605

[11] Saudi Press Agency. SFDA Approves Using Pfizer Vaccine To Age Category 5–11. Available online at: https://www.spa.gov.sa/viewfullstory.php?lang=en&newsid=2301189 (Accessed November 23, 2021).

[12] MacDonald NE. Vaccine hesitancy: definition, scope and determinants. Vaccine. (2015) 33:4161–4. 10.1016/j.vaccine.2015.04.036 25896383

[13] Ali M Hossain A. What is the extent of COVID−19 vaccine hesitancy in Bangladesh? BMJ Open. (2021) 11:e050303. 10.1136/bmjopen-2021-050303 34429316 PMC8387740

[14] Yasmin F Najeeb H Moeed A Naeem U Asghar MS Chughtai NU et al. COVID−19 vaccine hesitancy in the United States: a systematic review. Front Public Health. (2021) 9:770985. 10.3389/fpubh.2021.770985 34888288 PMC8650625

[15] Cerda AA García LY. Hesitation and refusal factors in individuals' decision–making processes regarding a Coronavirus disease 2019 vaccination. Front Public Health. (2021) 9. 10.3389/fpubh.2021.626852 PMC8096991 33968880

[16] Sallam M Dababseh D Eid H Al–Mahzoum K Al–Haidar A Taim D et al. High rates of COVID−19 vaccine hesitancy and its association with conspiracy beliefs: a study in Jordan and Kuwait among other Arab countries. Vaccines. (2021) 9:42. 10.3390/vaccines9010042 33445581 PMC7826844

[17] Sallam M. COVID−19 vaccine hesitancy worldwide: a concise systematic review of vaccine acceptance rates. Vaccines. (2021) 9:160. 10.3390/vaccines9020160 33669441 PMC7920465

[18] Alfieri NL Kusma JD. Heard–Garris N, Davis MM, Golbeck E, Barrera L et al. Parental COVID−19 Vaccine Hesitancy for Children: Vulnerability in an Urban Hotspot. BMC Public Health. (2021) 21:1662. 10.1186/s12889-021-11725-5 34517848 PMC8436579

[19] Bagateli LE Saeki EY Fadda M Agostoni C Marchisio P Milani GP. COVID−19 vaccine hesitancy among parents of children and adolescents living in Brazil. Vaccines. (2021) 9:1115. 10.3390/vaccines9101115 34696223 PMC8540804

[20] Musa S Dergaa I Abdulmalik MA Ammar A Chamari K Saad HB. BNT162b2 COVID−19 vaccine hesitancy among parents of 4023 young adolescents (12–15 Years) in Qatar. Vaccines. (2021) 9:981. 10.3390/vaccines9090981 34579218 PMC8473301

[21] Ali M Ahmed S Bonna A Sarkar A Islam M Urmi T et al. Parental coronavirus disease vaccine hesitancy for children in Bangladesh: a cross–sectional study. F1000 Research. (2022) 11:90. 10.12688/f1000research.76181.1 35211297 PMC8837808

[22] Horiuchi S Sakamoto H Abe SK Shinohara R Kushima M Otawa S et al. Factors of parental COVID−19 vaccine hesitancy: a cross sectional study in Japan. PLoS ONE. (2021) 16:e0261121. 10.1371/journal.pone.0261121 34919580 PMC8683027

[23] Altulaihi BA Alaboodi T Alharbi KG Alajmi MS Alkanhal H Alshehri A. Perception of parents towards COVID−19 vaccine for children in Saudi population. Cureus. (2021) 13:e18342. 10.7759/cureus.18342 34646710 PMC8481148

[24] Temsah M–H Alhuzaimi AN Aljamaan F Bahkali F Al–Eyadhy A Alrabiaah A et al. Parental attitudes and hesitancy about COVID−19 vs. routine childhood vaccinations: a national survey. Front Public Health. (2021) 9: 752323. 10.3389/fpubh.2021.752323 PMC8548678 34722451

[25] Daly M Jones A Robinson E. Public trust and willingness to vaccinate against COVID−19 in the US from October 14, 2020, to March 29, 2021. JAMA. (2021) 325:2397–9. 10.1001/jama.2021.8246 34028495 PMC8145162

[26] Carpenter CJ A. meta–analysis of the effectiveness of health belief model variables in predicting behavior. Health Commun. (2010) 25:661–9. 10.1080/10410236.2010.521906 21153982

[27] Rosenstock IM Strecher VJ Becker MH. Social learning theory and the Health Belief Model. Health Educ Q. (1988) 15:175–83. 10.1177/109019818801500203 3378902

[28] Bish A Michie S. Demographic and attitudinal determinants of protective behaviours during a pandemic: a review. Br J Health Psychol. (2010) 15:797–824. 10.1348/135910710X485826 20109274 PMC7185452

[29] Alobaidi S. Predictors of intent to receive the COVID−19 vaccination among the population in the kingdom of Saudi Arabia: a survey study. J Multidiscip Healthc. (2021) 14:1119–28. 10.2147/JMDH.S306654 34040382 PMC8140927

[30] Aldakhil H Albedah N Alturaiki N Alajlan R Abusalih H. Vaccine hesitancy towards childhood immunizations as a predictor of mothers' intention to vaccinate their children against COVID−19 in Saudi Arabia. J Infect Public Health. (2021) 14:1497–504. 10.1016/j.jiph.2021.08.028 34481723 PMC8390407

[31] Shmueli L. Parents' intention to vaccinate their 5–11 years old children with the COVID−19 vaccine: rates, predictors and the role of incentives (preprint). MedRxiv [Preprint]. (2021). 10.1101/2021.11.05.21265900 PMC9926441 36788509

[32] McElfish PA Willis DE Shah SK Reece S Andersen JA Schootman M et al. Parents&rsquo; and guardians&rsquo; intentions to vaccinate children against COVID−19. Vaccines. (2022) 10:361. 10.3390/vaccines10030361 35334993 PMC8949105

[33] Huynh G Nguyen HTN Van Tran K Le An P Tran TD. Determinants of COVID−19 vaccine hesitancy among parents in Ho Chi Minh city, Vietnam. Postgrad Med. (2022) 25:1–6. 10.1080/00325481.2022.2044142 35188041

[34] Li T Qiu X Gong X Zhan R Zheng X. The cross–sectional survey on COVID−19 vaccine hesitancy and it predictors among Chinese parents of 3–17 years aged children in Shenzhen city. Ann Agric Environ Med. (2022) 9:779720. 10.26444/aaem/146263 35352915

[35] Bianco A Della Polla G Angelillo S Pelullo CP Licata F Angelillo IF. Parental COVID−19 vaccine hesitancy: a cross–sectional survey in Italy. Expert Rev Vaccines. (2021) 2:1–7. 10.1080/14760584.2022.2023013 34949136

[36] Liz Hamel LL Grace Sparks Ashley Kirzinger Audrey Kearney Mellisha Stokes Mollyann Brodie. KFF COVID−19 Vaccine Monitor. (2021). Available online at: https://www.kff.org/coronavirus--covid$-$19/poll--finding/kff--covid$-$19--vaccine--monitor--october$-$2021/ (accessed November 28, 2021).

[37] Marquez RR Gosnell ES Thikkurissy S Schwartz SB Cully JL. Caregiver acceptance of an anticipated COVID−19 vaccination. J Am Dent Assoc. (2021) 152:730–9. 10.1016/j.adaj.2021.03.004 34059293 PMC7988472

