# Corrigendum: Parents' Hesitancy to Vaccinate Their 5–11-Year-Old Children Against COVID-19 in Saudi Arabia: Predictors From the Health Belief Model

**DOI:** 10.3389/fpubh.2022.914691

**Published:** 2022-06-01

**Authors:** Ohoud S. Almalki, Osamah M. Alfayez, Majed S. Al Yami, Yousif A. Asiri, Omar A. Almohammed

**Affiliations:** ^1^Department of Clinical Pharmacy, College of Pharmacy, Taif University, Taif, Saudi Arabia; ^2^Department of Pharmacy Practice, College of Pharmacy, Qassim University, Qassim, Saudi Arabia; ^3^Department of Pharmacy Practice, College of Pharmacy, King Saud bin Abdulaziz University for Health Sciences, Riyadh, Saudi Arabia; ^4^Department of Clinical Pharmacy, College of Pharmacy, King Saud University, Riyadh, Saudi Arabia; ^5^Pharmacoeconomics Research Unit, College of Pharmacy, King Saud University, Riyadh, Saudi Arabia

**Keywords:** vaccine, COVID-19, hesitancy, parents, Health Belief Model, Saudi Arabia

In the published article, there was an error in affiliation **1**. Instead of “*Department of Pharmacy Practice*,” it should be “*Department of Clinical Pharmacy*.”

Ohoud S. Almalki was mistakenly not credited as a contributing author in the original Author Contributions. The Author Contributions Statement previously stated:

OSA and OAA: conceptualization and project administration. OAA: methodology, software, data curation, formal analysis, and funding acquisition. OSA and MA: validation and writing—original draft preparation. OSA, OMA, and MA: investigation. YA, OMA, and OAA: writing—review and editing. OMA, MA, and YA: visualization. OSA, YA, and OAA: supervision. All authors have read and agreed to the published version of the manuscript.

The corrected Author Contributions Statement appears below.

## Author Contributions

OSA and OAA: conceptualization and project administration. OAA: methodology, software, data curation, formal analysis, and funding acquisition. OSA and MA: validation and writing—original draft preparation. OSA, OMA, and MA: investigation. YA, OMA, and OAA: writing—review and editing. OMA, MA, and YA: visualization. OSA, YA, and OAA: supervision. All authors have read and agreed to the published version of the manuscript.

The authors apologize for this error and state that this does not change the scientific conclusions of the article in any way. The original article has been updated.

## Publisher's Note

All claims expressed in this article are solely those of the authors and do not necessarily represent those of their affiliated organizations, or those of the publisher, the editors and the reviewers. Any product that may be evaluated in this article, or claim that may be made by its manufacturer, is not guaranteed or endorsed by the publisher.

