# Supplementary material for: Parents' Hesitancy to Vaccinate Their 5–11-Year-Old Children Against COVID-19 in Saudi Arabia: Predictors From the Health Belief Model
Source: Front Public Health. 2022 Mar 30;10:842862. doi: 10.3389/fpubh.2022.842862 (PMC9005777; doi:10.3389/fpubh.2022.842862)
Supplement: Supplementary file 1 [file Data_Sheet_1.PDF]

Questionnaire - الاستبيان

**First: General information about the participants**	**أولا: المعلومات العامة عن المشارك في الاستبيان**
1. **Age group:**<br>☐ 18 – 30 years<br>☐ 31 – 40 years<br>☐ 41 – 50 years<br>☐ 51 – 60 years<br>☐ Older than 60 years	١. **الفئة العمرية:**<br>☐ ١٨ – ٣٠ عام<br>☐ ٣١ – ٤٠ عام<br>☐ ٤١ – ٥٠ عام<br>☐ ٥١ – ٦٠ عام<br>☐ أكبر من ٦٠ عام
2. **Gender:**<br>☐ Male<br>☐ Female	٢. **الجنس:**<br>☐ ذكر<br>☐ أنثى
3. **Nationality:**<br>☐ Saudi<br>☐ Non-Saudi	٣. **الجنسية:**<br>☐ سعودي<br>☐ غير سعودي
4. **Marital status:**<br>☐ Single<br>☐ Married<br>☐ Separated<br>☐ Widow	٤. **الحالة الاجتماعية الحالية:**<br>☐ أعزب / عزباء<br>☐ متزوج / متزوجة<br>☐ منفصل / منفصلة<br>☐ أرمل / أرملة
5. **Highest educational qualification:**<br>☐ Less than high school<br>☐ High school<br>☐ Associate degree<br>☐ Bachelor’s degree<br>☐ Higher education (master’s degree or more)	٥. **أعلى مستوى تعليمي حصلت عليه:**<br>☐ أقل من ثانوي<br>☐ تعليم عام (ثانوي)<br>☐ دبلوم مهني<br>☐ جامعي (بكالوريوس)<br>☐ دراسات عليا (ماجستير فأعلى)
6. **Average annual income:**<br>☐ Less than $22,400<br>☐ $22,401 - $38,400<br>☐ $38,401 - $57,600<br>☐ $57,601 - $80,000<br>☐ More than $80,000	٦. **متوسط الدخل الشهري للأسرة:**<br>☐ أقل من ٧٠٠٠ ريال<br>☐ ٧٠٠١ - ١٢٠٠٠ ريال<br>☐ ١٢٠٠١ - ١٨٠٠٠ ريال<br>☐ ١٨٠٠١ - ٢٥٠٠٠ ريال<br>☐ أعلى من ٢٥٠٠٠ ريال
7. **Area of residence:**<br>☐ Riyadh<br>☐ Mecca<br>☐ Medina<br>☐ Eastern province<br>☐ Qassim<br>☐ Aseer<br>☐ Tabuk<br>☐ Hail<br>☐ Northern Borders province<br>☐ Al-Jawf<br>☐ Jazan<br>☐ Najran<br>☐ Al-Bahah	٧. **المنطقة التي تعيش فيها:**<br>☐ الرياض<br>☐ مكة المكرمة<br>☐ المدينة المنورة<br>☐ الشرقية<br>☐ القصيم<br>☐ عسير<br>☐ تبوك<br>☐ حائل<br>☐ الحدود الشمالية<br>☐ الجوف<br>☐ جازان<br>☐ نجران<br>☐ الباحة

**Second: Participant’s data that shows the status of previous SARS-CoV-2 infection and vaccination.**	**ثانيا: بيانات المشارك التي توضح حالة الإصابة بفيروس كورونا المستجد والحصول على اللقاح**
**Have you or an adult who lives with you been infected with the SARS-CoV-2 since the beginning of the pandemic?**<br>☐ Yes, myself or a family member is currently infected<br>☐ Yes, among us previously infected person/s who has recovered<br>☐ No, we were not infected with the virus	٨. **هل أصبت أنت أو أحد البالغين ممن يسكن معك في المنزل بفيروس كورونا المستجد منذ بداية الجائحة؟**<br>☐ نعم، أنا أو أحد البالغين في المنزل مصاب حالياً<br>☐ نعم، بيننا مصاب أو مصابين سابقين ومتعافين حالياً<br>☐ لا، لم نصب بفيروس كورونا
**Have any of your children been infected with the SARS-CoV-2 since the beginning of the pandemic?**<br>☐ Yes, one or more are currently infected<br>☐ Yes, previously infected, and recently recovered<br>☐ No, they were not infected	٩. **هل أصيب أي من أبناءك بفيروس كورونا المستجد منذ بداية الجائحة؟**<br>☐ نعم، مصاب أو عدة مصابين حالياً<br>☐ نعم، مصابين سابقين ومتعافين حالياً<br>☐ لا، لم يصابوا بفيروس كورونا
**Did you receive the COVID-19 vaccine, and how many doses did you receive?**<br>☐ Yes, two doses or more<br>☐ Yes, one dose<br>☐ No, did not receive any dose<br>☐ No, because I am from the exempted group	١٠. **هل تلقيت اللقاح الخاص بفيروس كورونا المستجد، وكم جرعة تلقيت؟**<br>☐ نعم - جرعتين أو أكثر<br>☐ نعم - جرعة واحدة<br>☐ لا - لم أتلقى أي جرعة من اللقاح<br>☐ لا - لأنني من الفئات المستثناة والمعفيين من شرط الحصول على اللقاح
**If you have children over the age of 12 and under the age of 18, have they had at least one dose of the COVID-19 vaccine?**<br>☐ I don't have children over the age of 12 years<br>☐ Yes, he/she have received two doses of the vaccine<br>☐ Yes, he/she have received one dose of the vaccine<br>☐ No, he/she did not receive any dose of the vaccine<br>☐ No, because he/she is from the exempted group	١١. **في حال كان لديك أبناء أكبر من ١٢ سنة ودون ١٨ سنة، هل حصلوا على الأقل على جرعة واحدة من اللقاح الخاص بفيروس كورونا المستجد؟**<br>☐ ليس لدي أبناء أكبر من ١٢ سنة<br>☐ نعم - تلقّى جرعتين من اللقاح<br>☐ نعم - تلقّى جرعة واحدة من اللقاح<br>☐ لا - لم يتلقّى أي جرعة من اللقاح<br>☐ لا - لأنه من الفئات المستثناة والمعفيين من شرط الحصول على اللقاح
**My main source of information on COVID-19 vaccine is:**<br>☐ TV<br>☐ Radio<br>☐ Social media<br>☐ Campaigns directed by the Ministry of Health in Saudi Arabia<br>☐ Health care provider<br>☐ Friends/relatives<br>☐ Other	١٢. **مصدر معلوماتي الرئيسي عن اللقاحات ضد فيروس كورونا المستجد هو:**<br>☐ التلفزيون<br>☐ الإذاعة<br>☐ وسائل التواصل الاجتماعي<br>☐ الحملات الموجهة للعامة من وزارة الصحة بالمملكة<br>☐ مقدم الرعاية الطبية<br>☐ الأصدقاء/الأقارب<br>☐ أخرى

Questionnaire - الاستبيان

**How would you rate the general health status of your children (5-11 years)?**<br>Very good<br>Good<br>Fair<br>Poor<br>Very poor	**١٣. كيف تقيم الصحة العامة لأطفالك (٥-١١ سنة)؟**<br>☐ جيد جدا<br>☐ جيدة<br>☐ مقبولة<br>☐ سيئة<br>☐ سيئة جدا
**Does any of your children (5-11 years) suffer from any chronic illness that requires them to take medication on a regular basis?**<br>Yes<br>No	**١٤. هل يعاني أحد أطفالك (٥-١١ سنة) من أي مرض مزمن يتطلب منهم تناول الأدوية بشكل مستمر؟**<br>☐ نعم<br>☐ لا
**I commit to giving my children (5-11 years)the annual seasonal influenza vaccine**<br>Yes<br>No	**١٥. التزم بإعطاء أطفالي (٥-١١ سنة) اللقاح السنوي ضد الإنفلونزا الموسمية؟**<br>☐ نعم<br>☐ لا
**If the new COVID-19 vaccine is available for children (5-11 years), will you take the initiative to vaccinate your children?**<br>Definitely yes<br>Probably yes<br>Probably no, I will wait for the majority of children to be vaccinated<br>Absolutely no, unless I have to or vaccination has become mandatory	**١٦. في حال توفر لقاح كورونا المستجد للأطفال (٥-١١ سنة)، هل ستبادر بتلقيح أبناءك (٥-١١ سنة)؟**<br>☐ قطعا نعم<br>☐ ربما نعم<br>☐ على الأرجح لا، سوف أنتظر حتى يتم تلقيح الأكثرية من هذه الفئة<br>☐ قطعا لا، إلا إذا كنت مضطراً لذلك أو أصبح التلقيح إلزامي

**Third: items related to the main aim of the study**					**ثالثا: العناصر الخاصة بالاستبانة الرئيسية في البحث**
**A- The following statements are related to the perceived susceptibility of contacting SARS-CoV-2 by your children (5-11 years):**					**أ - العبارات التالية تخص احتمالية إصابة أطفالك (٥-١١ سنة) بفيروس كورونا المستجد المتوقعة:**
	معارض بشدة<br>Strongly disagree	معارض<br>Disagree	موافق<br>Agree	موافق بشدة<br>Strongly agree	
17. The chance of my children getting the SARS-CoV-2 in the next few months is high.					١٧. إمكانية إصابة أطفالي بفيروس كورونا المستجد في الأشهر القليلة المقبلة عالية.
18. I am worried about the likelihood of my children getting the SARS-CoV-2.					١٨. أنا قلق من احتمال إصابة أطفالي بفيروس كورونا المستجد.
19. Getting the SARS-CoV-2 is a possibility for my children.					١٩. إصابة أطفالي بفيروس كورونا المستجد احتمال وارد بالنسبة لي.
**B- The following statements are related to the perceived severity of SARS-CoV-2 infection for one of your children (5-11 years):**					**ب - العبارات التالية تخص توقعات خطورة الإصابة بفيروس كورونا المستجد لأحد أطفالك (٥-١١ سنة):**
	معارض بشدة<br>Strongly disagree	معارض<br>Disagree	موافق<br>Agree	موافق بشدة<br>Strongly agree	
20. In general, complications from the SARS-CoV-2 are serious.					٢٠. بشكل عام، تعد مضاعفات فيروس كورونا المستجد خطيرة.
21. If one of my children gets infected with the SARS-CoV-2, he will be very sick.					٢١. إذا أصيب أحد اطفالي بفيروس كورونا المستجد، فسيكون مريضًا جدًا.
22. I am worried that my children will get the SARS-CoV-2.					٢٢. أخشى من إصابة اطفالي بفيروس كورونا المستجد.
**C- The following statements are related to the perceived benefits from the COVID-19 vaccine:**					**ج- العبارات التالية تخص الفوائد المتوقعة للقاح فيروس كورونا المستجد:**
	معارض بشدة<br>Strongly disagree	معارض<br>Disagree	موافق<br>Agree	موافق بشدة<br>Strongly agree	
23. Vaccination is a good idea because I will not have to worry about my children catching the SARS-CoV-2.					٢٣. اللقاح فكرة جيدة لأنني لن أقلق بشأن إصابة اطفالي بفيروس كورونا المستجد.
24. Vaccination decreases the chance of my children getting the SARS-CoV-2 or its complications.					٢٤. اللقاح يقلل من احتمالية إصابة اطفالي بفيروس كورونا المستجد ومضاعفاته.
**D- The following statements are related to the perceived barriers to receiving the COVID-19 vaccine:**					**د- العبارات التالية تخص العوائق المتوقعة من تلقي لقاح فيروس كورونا المستجد:**
	معارض بشدة<br>Strongly disagree	معارض<br>Disagree	موافق<br>Agree	موافق بشدة<br>Strongly agree	
25. The possible side effects from receiving the COVID-19 vaccine would interfere with my children's usual activities.					٢٥. الأثار الجانبية المحتملة بعد تلقي اللقاح ستؤثر على حياة أطفالي اليومية.
26. I am concerned about the efficacy of the COVID-19 vaccine.					٢٦. انا قلق على اطفالي من عدم فعالية اللقاح ضد فيروس كورونا.
27. I am concerned about the safety of COVID-19 vaccine					٢٧. انا قلق على اطفالي بشأن سلامة اللقاح ضد فيروس كورونا.
28. I am concerned about the fake/faulty COVID-19 vaccine.					٢٨. انا قلق بشأن تلقي اطفالي لقاح مزيف او خاطئ ضد فيروس كورونا.
**E- The following statements are related to the cues to cation for receiving the COVID-19 vaccine:**					**هـ - العبارات التالية تخص الدوافع لتلقي اللقاح:**
	معارض بشدة<br>Strongly disagree	معارض<br>Disagree	موافق<br>Agree	موافق بشدة<br>Strongly agree	
29. I will register my children to receive the COVID-19 vaccine, if I was given adequate information.					٢٩. سوف أسجل اطفالي لتلقي اللقاح ضد فيروس كورونا المستجد إذا أعطيت معلومات كافية عنه.
30. I will register my children to receive the COVID-19 vaccine, if the vaccine is taken by many in the public.					٣٠. سوف أسجل اطفالي لتلقي اللقاح ضد فيروس كورونا المستجد إذا تم أخذ اللقاح من قبل الكثير من الناس.
